# Delivering Cancer Care During the COVID-19 Pandemic: Recommendations and Lessons Learned From ASCO Global Webinars

**DOI:** 10.1200/GO.20.00423

**Published:** 2020-09-30

**Authors:** Abdul Rahman Jazieh, Stephen L. Chan, Giuseppe Curigliano, Natalie Dickson, Vanessa Eaton, Jesus Garcia-Foncillas, Terry Gilmore, Leora Horn, David J. Kerr, Jeeyun Lee, Clarissa Mathias, Angélica Nogueira-Rodrigues, Lori Pierce, Alvaro Rogado, Richard L. Schilsky, Jean-Charles Soria, Jeremy L. Warner, Kazuhiro Yoshida

**Affiliations:** ^1^ King Saud bin Abdulaziz University for Health Sciences and King Abdullah International Medical Research Center, Riyadh, Saudi Arabia; ^2^ State Key Laboratory of Translational Oncology, Department of Clinical Oncology, The Chinese University of Hong Kong, Hong Kong; ^3^ Istituto Europeo di Oncologia, IRCCS and University of Milano, Milano, Italy; ^4^ Tennessee Oncology, Nashville, TN; ^5^ ASCO, Alexandria, VA; ^6^ Department of Oncology, Oncohealth Institute, Fundacion Jimenez Diaz University Hospital, Autonomous University, Madrid, Spain; ^7^ Vanderbilt University, Nashville, TN; ^8^ University of Oxford, Oxford, UK; ^9^ Samsung Medical Center, Seoul, Korea; ^10^ NOB/Grupo Oncoclinicas, Salvador, Brazil; ^11^ Brazilian Society of Medical Oncology and Federal University of Minas Gerais, Belo Horizonte, Brazil; ^12^ Rogel Cancer Center, Michigan Medicine, Ann Arbor, MI; ^13^ ECO Foundation for Excellence and Quality in Oncology, Madrid, Spain; ^14^ ASCO Association for Clinical Oncology, Alexandria, VA; ^15^ Gustave Roussy, Paris, France; ^16^ Department of Surgical Oncology, Gifu University, Graduate School of Medicine, Gifu, Japan

## Abstract

**PURPOSE:**

In response to the COVID-19 pandemic, the ASCO launched a Global Webinar Series to address various aspects of cancer care during the pandemic. Here we present the lessons learned and recommendations that have emerged from these webinars.

**METHODS:**

Fifteen international health care experts from different global regions and oncology disciplines participated in one of the six 1-hour webinars to discuss the latest data, share their experiences, and provide recommendations to manage cancer care during the COVID-19 pandemic. These sessions include didactic presentations followed by a moderated discussion and questions from the audience. All recommendations have been transcribed, categorized, and reviewed by the experts, who have also approved the consensus recommendations.

**RESULTS:**

The summary recommendations are divided into different categories, including risk minimization; care prioritization of patients; health care team management; virtual care; management of patients with cancer undergoing surgical, radiation, and systemic therapy; clinical research; and recovery plans. The recommendations emphasize the protection of patients and health care teams from infections, delivery of timely and appropriate care, reduction of harm from the interruption of care, and preparation to handle a surge of new COVID-19 cases, complications, or comorbidities thereof.

**CONCLUSION:**

The recommendations from the ASCO Global Webinar Series may guide practicing oncologists to manage their patients during the ongoing pandemic and help organizations recover from the crisis. Implementation of these recommendations may improve understanding of how COVID-19 has affected cancer care and increase readiness to manage the current and any future outbreaks effectively.

## INTRODUCTION

The COVID-19 pandemic has affected health care delivery throughout the world and overwhelmed health care systems.^1^ This crisis has interrupted health care delivery for many patients with cancer, who often require frequent visits and extensive utilization of the health care system to manage their disease and treatment complications. This vulnerable population faces an increased risk of severe COVID-19 infection and mortality, increased cancer burden due to uncontrolled tumor growth because of delayed cancer diagnosis, or interruption of treatment necessitated by severe acute respiratory syndrome (SARS) CoV-2 infection precautions, as well as the delay or interruption of their usual care for other medical problems.^2-5^

**Context**

**Key Objective**
The COVID-19 pandemic presented multiple major challenges to oncologists worldwide who struggled with unprecedented dilemmas related to unknown medical illness complicated by logistic challenges and information overload. ASCO launched a Global Webinar Series about COVID-19 and cancer care to help oncologists navigate through this crisis.
**Knowledge Generated**
Speakers in these webinars were experts from different geographical regions and clinical backgrounds who shared their first-hand experiences with the audience and provided recommendations on different aspects of managing patients with cancer and services during the pandemic and in the recovery phase. These recommendations were compiled, categorized, and summarized into this article to make it convenient for the readers to attain maximum benefit from these Webinars.
**Relevance**
This article may provide guidance for oncology clinical care, leadership, management, and research. The recommendations are adaptable to different practice settings.

The risks associated with the pandemic extend beyond the patients to the health care team members, who have suffered a higher rate of SARS CoV-2 infection because of the nature of their work. In addition, health care providers are at risk for burnout, exhaustion, and emotional well-being disorders. Many have competing pressures of managing childcare needs and other family responsibilities, in addition to their concerns about transmitting the infection to their family members.^6-9^

The pandemic has challenged the health care system. As SARS CoV-2 emerged as a new disease, there were many unknown factors and little evidence-based information to inform decision making. The amount of conflicting, at times poor-quality, information being released at a rapid pace has been overwhelming to health care professionals and administrators. Therefore, there is a need among the oncology community for professional recommendations from a reliable source to assess the rapidly unfolding information frequently and objectively and place it into context for the care of this unique patient population.^10^ ASCO, through the Coronavirus Resource Center, provides timely and frequently updated information to aid the global oncology community to care for their patients.^11^ In addition, ASCO developed a webinar series of virtual educational sessions to provide a platform for health care providers from around the world to share their experiences of cancer management during this global health care emergency.

This article provides a summary of the recommendations that have emerged from these webinars.

## METHODS

The ASCO Global Webinar Series was launched on April 14, 2020, with four 1-hour weekly sessions followed by a monthly session beginning June 30. Each webinar includes two or three speakers from different countries and regions of the world, including countries that were heavily affected by the pandemic. The speakers bring extensive, frontline, real-world experience in handling COVID-19 in patients with cancer. This diverse group has different oncology backgrounds, including medical oncology, radiation oncology, surgery, laboratory medicine, psychology, and research. The webinars consist of short, didactic talks followed by a moderated panel discussion. Participants join from all over the world and contribute to the discussions. The expert speakers share the current state of knowledge about managing cancer during the COVID-19 crisis. The initial webinars focused on managing the acute adjustments needed in cancer care delivery to respond to the COVID -19 pandemic and how to minimize risk to patients and health care providers. Subsequent webinars provided guidance to health care providers to cope with the potential surge of cancer care after the crisis and how to implement changes and modifications to their practice that go beyond the current pandemic.

The contents of these webinars were transcribed and reviewed. Common themes were identified and categorized and then reviewed by the authors of this article.

## RECOMMENDATIONS

The following themes emerged from the panel’s recommendations: (1) risk minimization, (2) patient care prioritization, (3) health care team management, (4) research management, (5) providing effective virtual care, and (6) recovery phase preparations.

### Recommendations on Minimizing Risk

There are general recommendations to reduce the risk of exposure to infection for both patients and health care team by implementing precautionary measures in different practice settings (Table 1). This can be achieved by reducing overcrowding and ensuring social distancing among both health care team members and patients, in addition to early recognition, triaging, and management of suspected cases.^12,13^

**TABLE 1:**
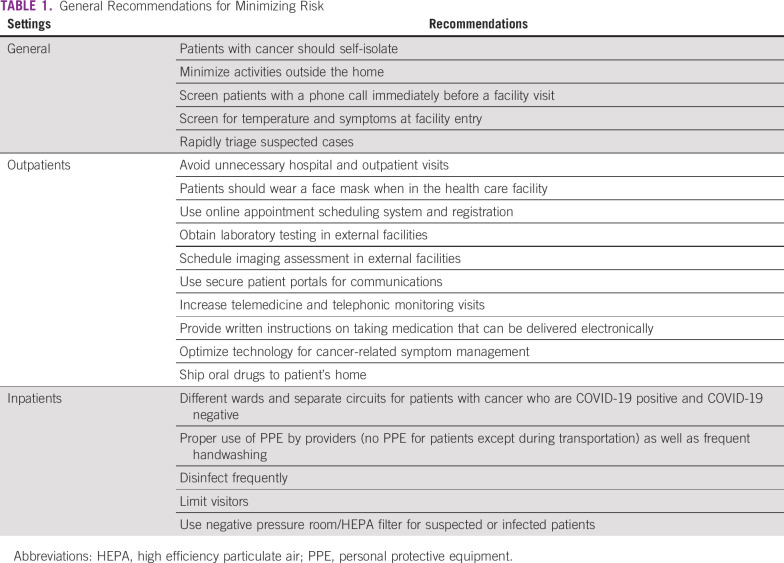
General Recommendations for Minimizing Risk

### Prioritization of Patient Care

There are multiple guidelines and recommendations for prioritizing patient care on the basis of the clinical condition of the patients and the status of the facility, based on the number of infected patients, available resources, and health of the workforce (Table 2).^14-17^

**TABLE 2:**
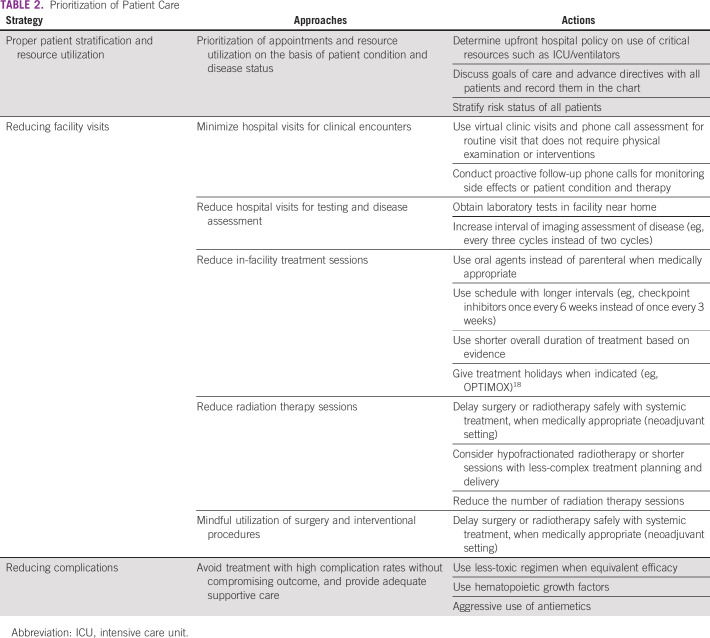
Prioritization of Patient Care

### Health Care Team Management and Protection

As mentioned earlier, health care workers are at increased risk for reasons that include infection, burnout, exhaustion, limited access to childcare and eldercare, as well as concerns about other family issues. The organization must implement precautions and measures to prevent infection and perform early detection and management of infected health care team members, in addition to providing support for their well-being, providing child/elder care alternatives, and developing a burnout mitigation system (Table 3).^19^

**TABLE 3:**
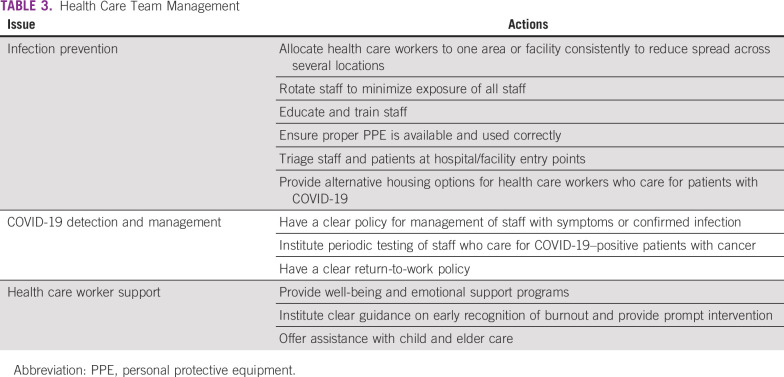
Health Care Team Management

Simple mitigation for burnout may require planning the distribution of work in advance, taking regular breaks, encouraging days off when appropriate, and seeking individual or program help. Although there are not any robust data on how to cope with burnout during COVID-19, a recently published article points to different programs that can be implemented to reduce health care team burnout.^20^

Among the factors that are necessary for health care professionals to stay healthy is the need to feel listened to, protected, and cared for. Medical societies and organizations may need to intervene to protect both the mental and physical health of their health care workers. They should also consider the need for preventive measures such as mindfulness, educational materials, in-the-moment measures such as hotlines and crisis support, treatment including telepsychiatry for therapy, and medication, if needed. It is critical for the health care worker to have access to and direct communication channels with organization administration and leaders to facilitate exchange of information and concerns in both directions.^7,9,21^

### Virtual Oncology and Telehealth

There are many potential applications for telemedicine in oncology care. The pandemic has resulted in the rapid expansion of telemedicine consultations to include urgent care, surveillance, new patients, hospital consultation, clinical trial consent and enrollment, genetic counseling, psychology, palliative care, follow-up, and survivorship.^22,23^

There is positive feedback from both patients and providers. However, there are still many challenges to optimize oncology care via telehealth. Organizations need to invest in appropriate infrastructure that includes adequate hardware and Internet bandwidth, training for providers regarding the optimal use of telehealth, and instruction on how to effectively communicate on a virtual platform. Patients also need to have access to mobile devices with audio-visual capabilities, Internet access, and the ability to navigate the technology. Interoperability between telemedicine software and other applications, such as practice management systems and electronic medical records, are required to facilitate scheduling and provide easily accessible medical records and documentation. Medical insurance coverage for this service is crucial for organizations and clinics to realize a return on investment. Furthermore, practitioner licensing and credentialing are necessary to provide virtual care across borders and should be addressed to facilitate better care and avoid provider liability.

If the provision of telehealth services is going to be available beyond the COVID-19 pandemic, it will require advo-cacy efforts through our specialty societies and through strengthened payer and government relations. Society should explore ways to ensure that all patients may benefit from this technology, especially those in underserved areas. There are still other opportunities for care that include easier access to second opinions, patient education about chemotherapy, and toxicity monitoring, as well as telepharmacy, care coordination, and nutrition counseling. Access to telemedicine allows clinics to reconsider staffing models and lease requirements.

### Management of Surgical Oncology

Surgical cancer care postponement requires careful consideration, because delaying diagnosis and definitive treatment could worsen oncological outcomes. In patients who are COVID-19 positive, surgical and anticancer medical care can lead to a worsening of their infection. Therefore, in patients with symptomatic infection, surgery should be limited to life-threatening situations until the patient has at least one negative test for COVID-19.^24^ However, requirements for surgery clearance may be modified, using caution to protect health care workers and hospital operation, based on individual cases considering the biology of each cancer, alternative treatment options, and waiting time for rescheduled surgery.^25^ Surgery on a COVID-19–positive patient can place providers and other hospital patients at risk. Intubation, extubation, mask ventilation, bronchoscopy, chest drainage placement, as well as electric scalpel treatment of digestive organs, laparoscopic surgery, and so on, can generate microscopic bubbles in the operating theater that can transmit infection. All patients must be tested before surgery, as asymptomatic people infected with SARS CoV-2 can shed and transmit the virus.

Head and neck oncologic surgery for malignancies of the upper aerodigestive tract represent a unique challenge during the COVID-19 pandemic; therefore, postponement or an alternative treatment should be considered. The hospital should focus on patients with symptoms requiring urgent surgery. In patients with GI, urological, or gynecologic cancer, surgery should be deferred in asymptomatic or minimally symptomatic cases (eg, benign and malignant polyps) and surgery considered only in emergency cases (eg, intestinal perforation secondary to cancer), significant symptomatic cases, and near-obstructing or cases with bleeding requiring large transfusion.

Preoperative polymerase chain reaction (PCR) test and/or chest computed tomography imaging can be useful tools for screening preoperative patients. In addition, a thorough history should be taken, including screening for symptoms, history of contact with infected persons, and behavior history before hospitalization for both the patient and their family, while restricting patient flow lines in hospitals. Limitations include personnel, inspection equipment, expendables (eg, personal protective equipment [PPE]), inclusion of cases that cannot be diagnosed even after screening, the reliability and accuracy of PCR, and cost.

Changes in postoperative care for oncology services include minimizing blood tests, scans, and routine tests to diminish risk of viral exposure, preferring oral anticancer drugs when possible, and shortening the duration of adjuvant chemotherapy.

### Clinical Research

Research activities face multiple challenges, including the lack of clinical staff and the ability to conduct trial activities and visits in many institutions. Clinical facilities in overwhelmed systems face revised use or lockdown and have not been able to perform elective procedures or admissions. Researchers must compete for prioritized resources, such as diagnostic tools, imaging, and laboratory analysis (Table 4).

**TABLE 4:**
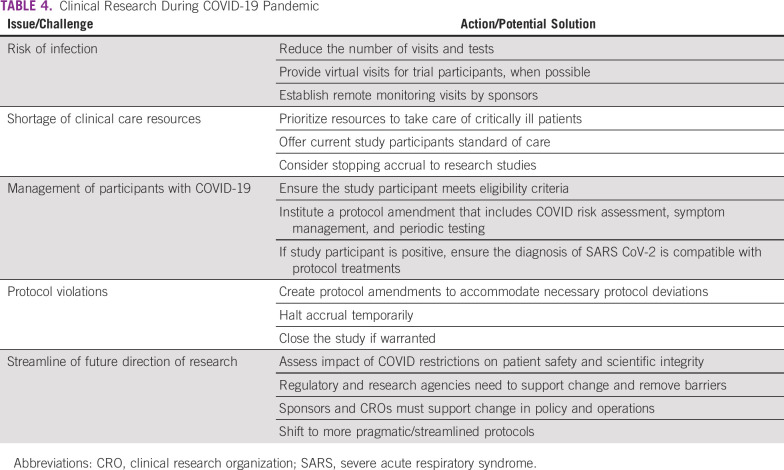
Clinical Research During COVID-19 Pandemic

Organizations have been prioritizing the safety of the participants as a primary concern and performing a risk/benefit assessment regarding the continuation of trials for both ongoing participants and the recruitment of new participants. The response has varied between halting recruitment or continuing with different approaches, to maintaining trial access and recruitment.^26,27^

Trial sponsors are concerned that access to staff, facilities, and participants is likely to be severely affected during the crisis time, and routine follow-up and monitoring activities are likely to be affected by investigator and staff availability and social distancing rules. Therefore, minimizing data collection or abstraction to what is critical to inform the primary end point is necessary during the crisis, as is the support for critical electronic systems, such as interactive response tools to manage the documentation of protocol deviations and so on.

Remote monitoring of electronic health records is essential and requires explicit instructions by the host and sponsors about what data are available and how to facilitate access from outside the facility. Trial participants need to consent to any identifiable health information, leaving the sites to ensure that confidentiality is protected. Safety monitoring visits must change substantially during the crisis, using phone calls to follow up on participants’ situations.^28^

The COVID-19 pandemic has forced hundreds of clinical trials to stop, stalling progress for cancer research. Patients with cancer sometimes consider their participation in trials to be crucial, and some participate to get new and otherwise unavailable treatment; however, there are also individuals who cannot tolerate available treatments and need access to alternative options or experimental drugs because of adverse effects. For now, it is unclear what long-term effects the pandemic will leave. The major shift in the way care is provided and research is conducted creates a huge opportunity for large collaborative studies and enhanced accrual of certain participants. In addition, the changing landscape creates multiple opportunities to do research in different ways and to participate in large studies that have surfaced because of the crisis.^29,30^

### Recommendations From Different Regions and Disciplines

The guiding principle of delivering care during the COVID-19 pandemic is to do so in a safe environment for patients and the health care team, prioritizing treatment of patients with curative intent, as well as providing for those in need of symptomatic palliation. Patient care should be prioritized to balance the risk of COVID-19 disease and the underlying cancer condition (ie, early-stage *v* late-stage disease). Treatment needs to be tailored to the individual, and, when possible, plans should be simplified to minimize the number of required in-person health care visits. For patients receiving radiotherapy, abbreviated fractionation schemes should be considered to reduce the time to deliver radiotherapy and potential viral exposure.^31,32^ For those cancers whose treatment course could be delayed with neoadjuvant systemic therapy, the pros and cons of delaying surgery and radiotherapy should be discussed.

Communication and coordination of care remains a priority before resuming normal hospital activities. Therefore, scheduling the patients previously postponed for screening or treatment should take priority.^33,34^

Patients who are experiencing anxiety regarding the safety of being treated must be reassured as they transition between different diagnostic and therapeutic services, such as radiology, pathology, surgery, radiation oncology, and medical oncology.

Frequent updates of COVID-19 guidelines for oncology patients are crucial for optimized cancer care. Treatment protocols for COVID-19–infected patients via multidisciplinary conferences with infectious disease specialists, oncologists, and/or governmental officials are critical to minimize unnecessary hospital visits, receive proper cancer care, and participate in clinical trials.

Organizations and clinicians must maintain virtual encounters and balance a remote and onsite workforce while gradually increasing the ability to perform complex treatment planning and deliver care. Treatment protocols for patients with SARS CoV-2 need to be further refined, exposure to the health care team must be reduced, and patients with cancer must be re-engaged with clinical research opportunities. This can be done safely through physical distancing measures and virtual visits.

It is critical to have a clear recovery plan as an extension of the crisis management plan, strong leadership, and communication to ensure well-coordinated care management (Table 5).^22^

**TABLE 5:**
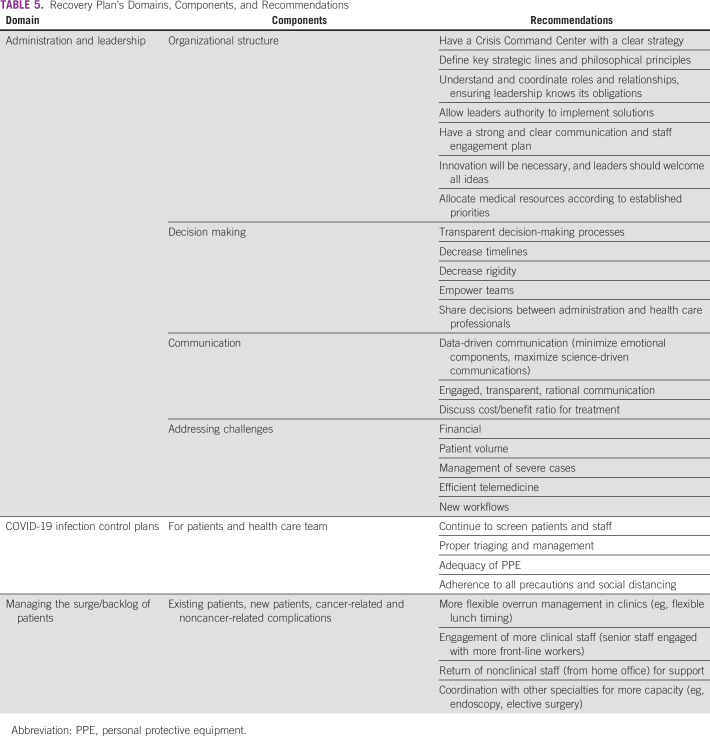
Recovery Plan’s Domains, Components, and Recommendations

We must remain vigilant against COVID-19 during the recovery phase by careful screening of patients and health care team, with proper triaging and management of individuals with suspected infections in addition to maintaining adequate supplies of PPE with appropriate distribution. For example, Korea experienced the first wave of COVID-19 early in February 2020, which appears to be controlled at the time of this writing; however, most Korean tertiary hospitals implemented virtual and on-site previsit surveillance programs to minimize the risk of asymptomatic or symptomatic COVID-19–infected individuals entering the hospital. The virtual and on-site previsit surveillance program for all patients and medical staff is updated every day, incorporating nationwide contact tracing information, which has been posted every morning in Korea. Individuals who have a history of contact with COVID-19–infected individuals or places (eg, restaurants, schools) are tested for SARS CoV-2 before they can enter any hospitals. In Italy, a hub-and-spoke model has been created, concentrating patients with cancer in hub hospitals and patients with COVID-19 in spoke hospitals. In the recovery phase, many countries require individuals to wear facial masks, especially in public areas, to prevent a resurgence of COVID-19; in addition, many countries are establishing recovery phase guidelines, according to their experience and needs.

**FIG 1:**
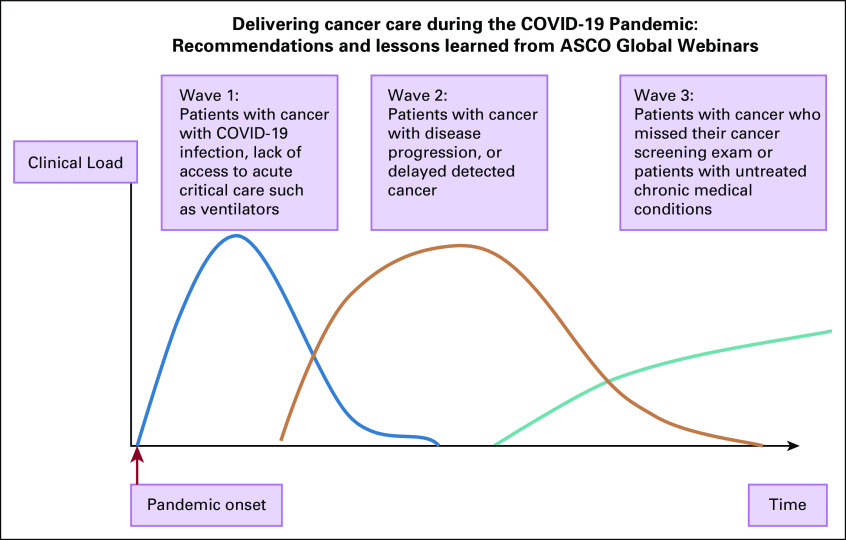
Medical care needs for patients with cancer during and post COVID-19 pandemic. Adapted and modified from Tapper and Asrani.^42^

A backlog of deferred follow-up cases and delays in new diagnosis of cancer will need to be managed, and the oncologist can expect increased complexities resulting from delay of treatment and/or disease progression and the patient’s comorbidities (Fig 1).

### Collaborative Efforts to Address a Common Challenge

This pandemic has been a catalyst for large-scale collaborative initiatives to understand the impact of the pandemic, as well as the management and outcome of patients with cancer during the pandemic. Some of these projects were conceived, developed, launched, and published in record time, highlighting the strong enthusiasm of the oncology community to collaborate in the face of the pandemic and the interest in learning in a timely fashion.

For example, the COVID-19 and Cancer Consortium (#CCC19) progressed from an idea to full peer-reviewed publication in 10 weeks.^35,36^ Now, with > 4,000 patients enrolled, the investigators will learn not just about the pattern of disease manifestations in patients with cancer, outcomes, and prognostic factors but also about the treatment of the infection, and they are looking at special subpopulations, such as disease type in the elderly.^37^ On the other hand, the TERAVOLT study is a global consortium with focus on thoracic cancers.^38^ This population represents a major challenge, as the virus mainly affects the lungs, with many overlapping features such as symptoms and imaging abnormalities. The study of the initial 400 patients revealed mortality of 35%, with older age, worse performance status, steroids before COVID-19 diagnosis, and active chemotherapy as predictors of worse outcome.

ASCO launched multiple projects related to the pandemic and cancer care. In a survey to assess the impact of the pandemic on clinical trials, ASCO captured the changes and challenges occurring in clinical research and the interventions implemented to mitigate the negative impact. The ASCO COVID-19 Registry was also launched to determine the distribution of symptoms and severity of COVID-19 among people with cancer, examine the impact of COVID-19 on cancer treatment and outcomes, and document adaptations of cancer care delivery due to the pandemic.^39^

Finally, mining CancerLinQ for COVID-19 cases among patients with cancer will be a source of real-world knowledge about how it is being managed and the disease outcomes.^40^

## DISCUSSION

The ASCO Global Webinar Series illustrates the importance of adopting new approaches to address emergent challenges in health care by delivering educational activities that are based on the society members’ needs and the current circumstances. Webinars were well received, as evident by the number of attendees from different countries and regions for both the live and recorded video sessions. Between April 14 and May 19, webinar recordings generated 1,311 total views from 33 countries.

Virtual webinars have been a widely used tool for education during the various phases of the pandemic, with many societies and organizations using them to reach their audiences. The approach by ASCO is distinguished by using international experts with wide experience in the management of cancer, as well as COVID-19, bringing diversity in terms of disciplines, background, and health care settings. The webinars reached a wide audience of ASCO members and were supported by the infrastructure of the Society and related activities such as the Coronavirus Resource Center, as well as the ASCO Survey on COVID-19 in Oncology Registry (ASCO Registry). In the absence of in-person scientific meetings for the foreseeable future, this type of technology fosters a sense of ongoing community and enables distance learning, including the acquisition of continued medical education credits.

However, there are still many challenges ahead in the fight against COVID-19, with many unanswered questions facing practicing oncologists on a daily basis. Although the recommendations provided in these webinars and summarized herein are based on the wide experience of different centers and the known literature, many are empirical and need validation with the test of time and appropriate prospective research studies. Reflecting on real-world experience with data collected in the large registries that have been created will also help us extrapolate and determine what is the best evidence to be used in managing future cases of COVID-19 outbreaks or other pandemics. Differences in the prevalence of COVID-19 cases, clinical settings, staffing, and access to resources will result in varying adoption of many of these recommendations. Collective wisdom and experience from different organizations, communities, and governments will help shape the best approach to control the pandemic at a global level. This, in turn, will positively affect all patients, including patients with cancer.^41^

The recommendations derived from ASCO Global Webinars have practical implications to oncology care to help manage patients and health care teams during the COVID-19 pandemic. The implementation of these recommendations should be adapted based on the practice setting, the pandemic’s evolution, and current state of knowledge.
